# Non-standard pipeline without MRI has replicability in computation of Centiloid scale values for PiB and ^18^F-labeled amyloid PET tracers

**DOI:** 10.1016/j.ynirp.2022.100101

**Published:** 2022-05-21

**Authors:** Motonobu Fujishima, Hiroshi Matsuda, for the Alzheimer's Disease Neuroimaging Initiative

**Affiliations:** a Department of Radiology, Kumagaya General Hospital, 4-5-1 Nakanishi, Kumagaya, Saitama, 360-8567, Japan; b Department of Biofunctional Imaging, Fukushima Medical University, 1 Hikariga-oka, Fukushima, 960-1295, Japan; c Drug Discovery and Cyclotron Research Center, Southern Tohoku Research Institute for Neuroscience, 7-61-2 Yatsuyamada, Koriyama, Fukushima, 963-8052, Japan; d Department of Radiology, National Center of Neurology and Psychiatry, 4-1-1 Ogawahigashi, Kodaira, Tokyo, 187-8551, Japan

**Keywords:** Centiloid, Pittsburgh compound B, Florbetapir, Flutemetamol, Florbetaben, NAV4694

## Abstract

The magnetic resonance imaging (MRI)-less non-standard pipeline for amyloid positron emission tomography (PET) published by Bourgeat et al., in 2018 calculates Centiloid scale values that are highly consistent with those computed using the standard pipeline. The purpose of this study was to demonstrate that the non-standard pipeline can compute Centiloid scale values in high agreement with the standard pipeline when using different datasets of amyloid PET tracers in our local computer environment.

PET images of ^11^C-Pittsburgh compound B (^11^C-PiB), ^18^F-florbetapir, ^18^F-flutemetamol, ^18^F-florbetaben, and ^18^F-NAV4694 from the calibration dataset were processed using both the standard and non-standard pipelines, and the computed cortical standardized uptake value ratio (SUVr) value was converted to the Centiloid scale value using the method described by Klunk et al., in 2015. The conversion equations from the SUVr to Centiloid scale values for each tracer were obtained during this process. Using these equations, we compared the Centiloid scale values obtained using the standard and non-standard pipelines using the validation datasets of each tracer from the Japanese Alzheimer’s Disease Neuroimaging Initiative (J-ADNI), Alzheimer’s Disease Neuroimaging Initiative (ADNI), and Australian Imaging Biomarkers and Lifestyle (AIBL).

In the calibration datasets, there was high agreement (R^2^ > 0.97) and slight bias between the Centiloid scale values calculated by the non-standard and standard pipelines for all tracers. Despite relatively little NAV4694 data in the validation datasets, there was high agreement between the Centiloid scale values calculated using the non-standard and standard pipelines for all tracers. The bias for florbetaben and NAV4694 using the non-standard pipeline was 1.6% underestimation and 3.3% overestimation, respectively; these values were smaller than those reported by Bourgeat et al. Analysis of outliers also suggested that the non-standard pipeline might be vulnerable to anatomical anomalies. Given the slight variance of the Centiloid scale in young controls, flutemetamol and NAV4694 might be suitable tracers for the non-standard pipelines.

This study demonstrates the replicability of the non-standard pipelines across computing environments, datasets, scanners, and tracers. When MRI is not available, the non-standard pipeline may provide information to aid in visual assessment of amyloid PET.

## Highlights

• The pipeline by Bourgeat et al. was replicable for computation of Centiloid scale values.
• In addition to PiB, FMM and NAV might be suitable for this non-standard pipeline.
• This pipeline may be vulnerable to anatomical anomalies.
• This pipeline may assist in the visual assessment of amyloid PET.

## 1 Introduction

Alzheimer's disease (AD) is a progressive disease that accounts for 60–80% of all dementia cases and causes degeneration and atrophy of the hippocampus and cerebral cortex (Alzheimer’s Association, 2021). AD often develops in old age, is associated with memory problems, and progresses slowly. Histopathologically, AD is characterized by extracellular senile plaques with amyloid-β (Aβ) as the major component and intracellular neurofibrillary tangles of which one of the major components is phosphorylated tau (Hyman et al., 2012). The amyloid cascade hypothesis starts with an increase in the production of Aβ monomers, followed by oligomerization of Aβ, protofibrils, and insoluble fibrils, leading to the formation of Aβ plaques, diffusion of phosphorylated tau, neuronal loss, and finally, to the clinical symptoms of cognitive decline. This hypothesis has been the mainstream theory of AD pathophysiology (Hampel et al., 2021). Aducanumab, the first disease-modifying drug for AD approved by the US Food and Drug Administration (FDA) in 2021, and the other humanized monoclonal antibodies against Aβ, namely, lecanemab, donanemab, and gantenerumab, were designed based on this hypothetical model.

More recently, an expert panel published recommendations for the appropriate use of aducanumab (Cummings et al., 2021). They recommended that its use be limited to patients with amyloid positron emission tomography (PET) positivity or findings consistent with AD on cerebrospinal fluid examination and with mild cognitive impairment (MCI) or mild dementia.

Amyloid PET tracers, such as ^11^C-Pittsburgh compound B (PiB), bind to Aβ plaques and can image and estimate the density of Aβ plaques in the brain. This has been demonstrated in the ^18^F-labeled amyloid PET tracers, ^18^F-florbetapir (FBP) (Clark et al., 2011), ^18^F-flutemetamol (FMM) (Curtis et al., 2015), and ^18^F-florbetaben (FBB) (Sabri et al., 2015), with binary visual assessment of in vivo PET and the Consortium to Establish a Registry for Alzheimer's Disease (CERAD) neuritic plaque scores (none or sparse vs. moderate or frequent neuritic plaques) on postmortem histopathological examinations, showing high sensitivity and specificity. ^18^F-NAV4694 (NAV) is an 18F-labeled amyloid PET tracer with low nonspecific binding to white matter and similar visual imaging characteristics to PiB in terms of visual assessment (Rowe et al., 2013). NAV demonstrated similar cortical-to-white matter ratios to PiB in healthy age-matched controls and subjects with AD dementia, and the ratio obtained with NAV in subjects with AD was higher than those obtained with other ^18^F-labeled amyloid PET tracers (Rowe et al., 2013).

Although the evaluation of amyloid PET is based on the binarization of positive/negative results by visual assessment (Cummings et al., 2021), the accuracy of visual assessment can be improved using a reader-independent semiquantitative evaluation method as an adjunct (Pontecorvo et al., 2017).

The Centiloid scale, published in 2015, is a method that outputs standardized, semiquantitative indices for any PET tracer or quantification method (Klunk et al., 2015). In this method, the Aβ accumulation level is scaled from 0 in healthy adults to 100 in those with typical AD dementia. Since the respective Centiloid scale thresholds for the CERAD neuritic plaque score and visual assessment have already been reported (Amadoru et al., 2020), the use of the Centiloid scale as an adjunct might contribute to increased accuracy of visual assessment at any institution, regardless of which tracer is used.

The Centiloid scale is calculated by anatomically normalizing the patient's PET image to the MNI-152 template space using the patient’s 3D T1-weighted magnetic resonance (MR) image with Statistical Parametric Mapping 8 (SPM8) using the volume of interest (VOI) of the whole cerebellum and that of the cerebral cortex (Klunk et al., 2015). We will refer to it as the "SPM8 standard pipeline". Multiple magnetic resonance imaging (MRI) examinations are recommended for patients who are candidates for aducanumab both before and during treatment to monitor for side effects, namely, amyloid-related imaging abnormalities (ARIA) (Cummings et al., 2021). However, if, for example, an MRI scan performed at another facility is not available at the time of PET analysis, or if the MRI scan performed does not include a 3D T1-weighted image, the Centiloid scale cannot be calculated with the SPM8 standard pipeline. Bourgeat et al. presented a non-standard approach to compute the Centiloid scale (Bourgeat et al., 2018) using a method to quantify PET standardized uptake value ratio (SUVr) without MRI called Computational Analysis of PET from the Australian Imaging, Biomarker & Lifestyle Flagship Study of Ageing (CapAIBL) (Bourgeat et al., 2015). We will refer to it as the "non-standard pipeline" hereafter.

CapAIBL (https://milxcloud.csiro.au/) is a cloud-based application, and the number of uses is limited to 10 images per day and a total of 100 images. Suppose the analysis can be performed on a local workstation. In that case, this application could be used without being limited by the number of images and without considering the risk of information leakage when transferring the patient's image data online. The purpose of this study was to replicate the non-standard Centiloid scale computation pipeline proposed by the original paper (Bourgeat et al., 2018) in our local environment and to investigate whether it could provide a high agreement for the Centiloid scale with the SPM8 standard pipeline using validation datasets consisting of PiB, FBP, FMM, FBB, and NAV.

## 2 Materials and methods

### 2.1 Participants

Data used for the level 2 analysis recommended by the Centiloid project paper (Klunk et al., 2015) were obtained from the Global Alzheimer's Association Interactive Network (GAAIN; http://www.gaain.org/centiloid-project). Data used for the validation of the non-standard procedure were drawn from the Australian Imaging Biomarkers and Lifestyle (AIBL; http://www.aibl.csiro.au/) (Ellis et al., 2009), the Japanese Alzheimer’s Disease Neuroimaging Initiative (J-ADNI; https://humandbs.biosciencedbc.jp/en/hum0043-v1) (Iwatsubo et al., 2018), and the Alzheimer’s Disease Neuroimaging Initiative (ADNI; adni.loni.usc.edu). The ADNI was launched in 2003 as a public-private partnership, led by principal investigator Michael W. Weiner, MD. The primary goal of ADNI has been to test whether serial MRI, PET, other biological markers, and clinical and neuropsychological assessment can be combined to measure the progression of MCI and early AD. The demographic data from each dataset are exhibited in Table 1.

**Table 1:** Demographics.

Dataset	N	Clinical group
For the level 2 analysis		
GAAIN PiB^a^	79	34 YCs, 45 AD
GAAIN PiB/FBP^a^	46	13 YCs, 6 OHCs, 3 at risk, 7 MCI, 3 possible AD, 14 AD
GAAIN PiB/FMM^a^	74	24 YCs, 10 OHCs, 20 MCI, 20 AD
GAAIN PiB/FBB^a^	35	10 YCs, 6 OHCs, 9 MCI, 8 AD, 2 FTD
GAAIN PiB/NAV^a^	55	10 YCs, 25 OHCs, 10 MCI, 7 AD, 3 FTD
For the validation analysis		
J-ADNI PiB group A	78	29 OHCs, 34 MCI, 15 AD (44 PiB-positive)
J-ADNI PiB group B	78	24 OHCs, 27 MCI, 27 AD (44 PiB-positive)
ADNI FBP	484	227 OHCs, 51 SMC, 78 early MCI, 72 MCI, 35 late MCI, 21 AD
AIBL FMM^b^	195	133 OHCs, 2 reversion from MCI to OHC, 43 MCI, 16 AD, 1 other dementia
ADNI FBB	323	176 OHCs, 1 early MCI, 108 MCI, 1 late MCI, 37 AD
AIBL NAV^b^	25	17 OHCs, 7 MCI, 1 AD

Abbreviations: AD, Alzheimer’s disease; FTD, frontotemporal dementia; MCI, mild cognitive impairment; OHCs, older healthy controls; SMC, significant memory concern; YCs, young controls.

a The same dataset as the original paper.

b Part of the same dataset as the original paper. We downloaded pairs of FMM PET scans and corresponding MRI scans and pairs of NAV PET scans and corresponding MRI scans used in the AIBL study from the ADNI site. As of December 3, 2021, considerably fewer pairs of PET and MRI scans were available through the ADNI site than those used in the original paper.

**Table 2:** Conversion equations from the SUVr values of^18^F-labeled tracers to PiB-equivalent SUVr and R^2^ values of the correlation between the PiB-equivalent SUVr and PiB SUVr values.

Tracer	SPM8 standard pipeline		Non-standard pipeline	
	SPiB−CalcUVrIND	$R^{2}$^∗^	SPiB−CalcUVrIND	$R^{2}$^†^
FBP	SFBPUVrIND−0.5110.523	0.905	SFBPUVrIND−0.6410.462	0.879
FMM	SFMMUVrIND−0.2180.770	0.963	SFMMUVrIND−0.3470.703	0.965
FBB	SFBBUVrIND−0.3900.608	0.954	SFBBUVrIND−0.4900.557	0.957
NAV	SNAVUVrIND+0.0351.046	0.991	SNAVUVrIND−0.1450.899	0.990

∗SPiB−CalcUVrIND vs. SPiBUVrIND

†SPiB−CalcUVrIND vs. SPiBUVrIND

### 2.2 SPM8 standard pipeline

#### 2.2.1 Replication of the level 1 analysis

To demonstrate that the SPM8 standard pipeline proposed in the Centiloid project paper can accurately calculate the Centiloid scale in our local environment, the level 1 analysis described in Section 2.2.2.1. of the Centiloid project paper was reproduced as part of the level 2 analysis. The 50–70-min dataset for calibration of the standard PiB method consisting of 34 healthy young subjects (YC-0 subjects) and 45 typical AD dementia subjects (AD-100 subjects) was downloaded from the GAAIN website. First, each subject's PiB PET image was rigidly registered to the MNI-152 template implemented in SPM8 using the symmetric version of reg_aladin implemented in NiftyReg (Modat et al., 2014; Ourselin et al., 2001) rather than by manual reorientation to the template. Note that the resultant transformation was not used to resample the PET image but to update the sform stored in the NIfTI header of the PET image. From this point on, SPM8 was used for the standard pipeline. The PiB PET image was then rigidly co-registered to the same subject's MRI (3D T1-weighted image). The 3D T1-weighted image of each subject was anatomically normalized to the MNI-152 template, and the PiB PET image co-registered to the 3D T1-weighted image was anatomically normalized to the MNI-152 space using the resultant forward normalization parameter. The whole cerebellar VOI of voxel size 2 × 2 × 2 mm^3^ downloaded from the GAAIN website was used as the reference region, and the cortical VOI of voxel size 2 × 2 × 2 mm^3^ was used to calculate the SUVr of the spatially normalized PiB PET image. The SUVr was then converted to the Centiloid (CL) scale using the following Equation (1) (cited from Equation 2.2.1 of the Centiloid project paper):

(1) CL=100×SPiBUVrIND∗−SPiBUVrYC−0∗SPiBUVrAD−100∗−SPiBUVrYC−0∗

where SPiBUVrIND∗ denotes the SUVr of an individual subject, SPiBUVrYC−0∗ denotes the mean SUVr of the 34 YC-0 subjects, and SPiBUVrAD−100∗ denotes the mean SUVr of the 45 AD-100 subjects. Finally, linear regression analysis was performed using the resultant Centiloid scale values as the independent variable and those reported in the Centiloid paper as the dependent variable to calculate the slope, intercept, and coefficient of determination R^2^.

#### 2.2.2 Level 2 calibration of ^18^F-labeled tracers

In this section, ^18^F-labeled tracer PET was calibrated into the Centiloid scale based on PiB. The PiB/FBP dataset (Navitsky et al., 2018), PiB/FMM dataset (Battle et al., 2018), PiB/FBB dataset (Rowe et al., 2017), and PiB/NAV dataset (Rowe et al., 2016) were downloaded from the GAAIN website and then analyzed. The SUVr was calculated from the PiB PET image and the corresponding MRI from each dataset by the standard pipeline described in 2.2.1. and then converted to the Centiloid scale using Equation (1). Similarly, the SUVr was calculated from an ^18^F-labeled tracer PET image and the corresponding MRI. Next, linear regression analysis was performed with the SUVr of the PiB PET image as the independent variable and that of the ^18^F-labeled tracer PET image as the dependent variable using the following Equation (2) (cited from Equation 2.2.3.1a of the Centiloid project paper):

(2) STracerUVrIND=mTracerStd×(SPiBUVrIND∗∗)+bTracerStd

where SPiBUVrIND∗∗ denotes the PiB PET SUVr of an individual subject from the GAAIN datasets as abovementioned. The resultant slope (mTracerStd) and intercept (bTracerStd) were used to calculate the PiB-equivalent SUVr (SPiB−CalcUVrIND) from the SUVr of the ^18^F-labeled tracer PET image (STracerUVrIND) using the following Equation (3) (cited from Equation 2.2.3.1b in the Centiloid project paper):

(3) SPiB−CalcUVrIND=STracerUVrIND−bTracerStdmTracerStd

Finally, the Centiloid scale of the ^18^F-labeled tracer PET image was calculated using the following Equation (4) (cited from Equation 2.2.3.1c in the Centiloid project paper):

(4) CTracerLStd=100×SPiB−CalcUVrIND∗∗−SPiBUVrYC−0∗SPiBUVrAD−100∗−SPiBUVrYC−0∗

#### 2.2.3 Validation using the datasets from J-ADNI, ADNI, and AIBL

Independent validation datasets were drawn from J-ADNI for PiB, ADNI for FBP and FBB, and AIBL for FMM and NAV. The FMM and NAV datasets were from AIBL as in the original paper. However, there were fewer subjects than in the original paper (195 and 25 subjects in the FMM and NAV datasets, respectively, versus 446 and 79 subjects, respectively) because fewer AIBL subjects had both FMM and NAV data available on the ADNI website than in the original paper. The other datasets were taken from studies that were markedly different from the original paper.

These datasets were analyzed using the SPM8 standard pipeline to compute the SUVr. For an individual PiB PET image, the SUVr (SPiBUVrIND) was converted into the Centiloid scale using Equation (1). For an individual ^18^F-labeled PET tracer PET image, the SUVr (STracerUVrIND) was first converted into the PiB-equivalent SUVr (SPiB−CalcUVrIND) using Equation (3) and finally converted into the Centiloid (CTracerLStd) scale using Equation (4). The Centiloid scale computed here was compared with that computed using the non-standard pipeline mentioned in Section 2.3.3 from the same validation datasets.

### 2.3 Non-standard pipeline — replication of the original paper (Bourgeat et al., 2018)

#### 2.3.1 Details of the non-standard pipeline

The non-standard pipeline for calculating the SUVr consists of the following three steps:

1) Creation of the PiB-negative and PiB-positive PET templates

CapAIBL requires an amyloid PET template to spatially normalize amyloid PET images without the corresponding structural MRI (Bourgeat et al., 2015). To separate the data used for template creation from those used for validation, we divided the 156 cases from the J-ADNI dataset, which consists of PiB and MRI combinations, into two groups (group A of n = 78 and B of n = 78) so that the amyloid negativity/positivity of the J-ADNI reading results were evenly distributed. Subsequent operations were performed separately for groups A and B. In each group, the MRI of each subject was spatially normalized to the J-ADNI structural template using linear and nonlinear registration of ANTs (Avants et al., 2008). This template originated from the one created in our previous study (Fujishima et al., 2017) and was resampled to a voxel grid of 160 × 160 × 96 with a voxel size of 1.5 × 1.5 × 1.5 mm^3^. The resultant normalization parameter was applied to the PiB PET image of the same participant. The PiB-negative PET template ($A_{neg}$) and PiB-positive template ($A_{pos}$) were created by averaging the spatially normalized PiB-negative images (n = 34 in group A and n = 34 in group B) and PiB-positive PET images (n = 44 in group A and n = 44 in group B), respectively.

2) Spatial normalization using the adaptive template

According to the original paper, “the adaptive template is a linear combination of an Aβ negative ($A_{neg}$) and Aβ positive ($A_{pos}$) template, with a weight w optimized by maximizing the normalized mutual information between the adaptive template and the target:”

(5) $$\begin{array}{l} A\left(w\right)=w\times A_{neg}+\left(1-w\right)\times A_{pos} \end{array}$$

where A(w) denotes the adaptive template. Each amyloid PET image was affinely registered to the mixed template generated by averaging the $A_{neg}$ and $A_{pos}$ templates using reg_aladin. Next, normalized mutual information was computed between the affinely registered PET image and A(w). The optimization was performed using Powell’s method (Powell, 1964) before nonlinear registration. The optimization was initialized with w=0.5 (Bourgeat et al., 2015). The nonlinear registration was performed using reg_f3d implemented in NiftyReg (Modat et al., 2010) with 20-mm control point grid spacing (Bourgeat et al., 2015).

3) Calculation of the SUVr

**Fig. 1:**
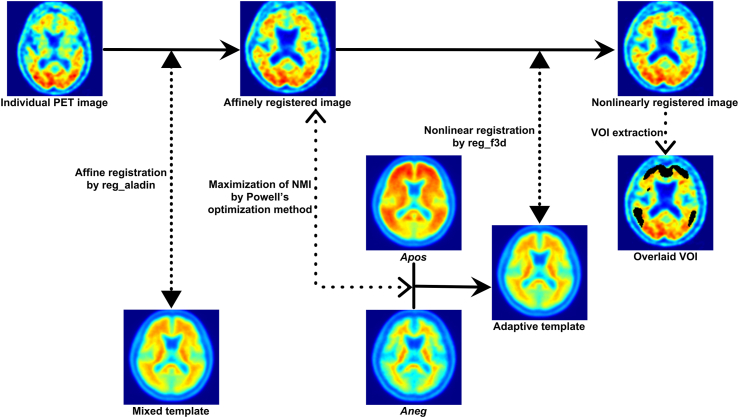
Flow chart of the non-standard pipeline. Each PET image is affinely registered to the mixed template using reg_aladin. The NMI is then computed between the affinely registered image and the adaptive template. The adaptive template is generated by linearly combining $A_{neg}$ and $A_{pos}$. The NMI is maximized using Powell’s optimization method. Finally, the affinely registered image is nonlinearly registered to the adaptive template using reg_f3d. The SUVr is computed using the cortical and whole cerebellar VOIs for the nonlinearly registered image. Abbreviations: $A_{neg}$, PiB-negative template; $A_{pos}$, PiB-positive template; NMI, normalized mutual information; VOI, volume of interest.

The cortical target VOI and whole cerebellar reference VOI in the MNI-152 space from the GAAIN website were deformed to the J-ADNI template space using the normalizing parameter computed by ANTs. The SUVr was calculated for the spatially normalized amyloid PET image using the deformed VOIs. The flow chart of the non-standard pipeline is exhibited in Fig. 1.

#### 2.3.2 Level 2 analysis of the non-standard pipeline using PiB and ^18^F-labeled tracers

Linear regression analysis was performed with the SUVr of the PiB PET image using the SPM8 standard procedure as described in Section 2.1 as the independent variable and that of the PiB or ^18^F-labeled PET image using the non-standard pipeline as described in Section 2.2.1 as the dependent variable using the following Equation (6) (cited from Equation 2.2.3.2a in the Centiloid project paper):

(6) STracerUVrIND=mTracerNS×(SPiBUVrIND∗∗)+bTracerNS

The resultant slope (mTracerNS) and intercept (bTracerNS) were used to convert the SUVr value using the non-standard pipeline (STracerUVrIND) into the PiB-equivalent SUVr (SPiB−CalcUVrIND) as expressed in the following Equation (7) (cited from Equation 2.2.3.2b in the Centiloid project paper):

(7) SPiB−CalcUVrIND=STracerUVrIND−bTracerNSmTracerNS

where the “NS” subscript denotes the non-standard pipeline. The Centiloid scale using the non-standard pipeline was calculated using Equation (4).

#### 2.3.3 Validation using independent datasets from J-ADNI, ADNI, and AIBL

The same validation datasets used in Section 2.2.3 were analyzed using the non-standard pipeline. The computed SUVr was converted into the PiB-equivalent SUVr (SPiB−CalcUVrIND) using Equation (7) and scaled into the Centiloid scale using Equation (4). The Centiloid scale values calculated using the non-standard pipeline in this section and the SPM8 standard pipeline in Section 2.2.3 were compared from the viewpoint of R^2^, slope of the regression, and intra-class correlation (ICC). The ICC was calculated using two-way mixed effect models and a single-rater unit based on absolute agreement.

## 3 Results

### 3.1 Replication of the level 1 analysis of the SPM8 standard pipeline

The mean SUVr (± standard deviation (SD)) of 34 YC-0 subjects (SPiBUVrYC−0∗) and 45 AD-100 subjects (SPiBUVrAD−100∗) were 1.014±0.047 and 2.088±0.209, respectively, and those differences were within 1% when compared with those in the level 1 analysis in the Centiloid paper. When a linear regression analysis was performed using the Centiloid scale values in our local environment and those reported in the Centiloid paper, the slope was 0.997, the intercept was 0.166, and R^2^ was 0.999, demonstrating that all the values were within the acceptable ranges (between 0.98 and 1.02 for the slope, between −2 and 2 for the intercept, and >0.98 for R^2^) (Klunk et al., 2015). By substituting the mean SUVr values (SPiBUVrYC−0∗ and SPiBUVrAD−100∗) into Equation (4), the PiB SUVr using the SPM8 standard pipeline was converted into the Centiloid scale using the following equation (Table 3):

CPiBLStd=100×SPiBUVrIND−1.0141.074

**Table 3:** Conversion equations from the SUVr values of each tracer to the Centiloid and R^2^ values of the correlation between the non-standard and SPM8 standard pipelines.

Tracer	SPM8 standard pipeline	Non-standard pipeline	*R*^2^*
	CTracerLStd	CTracerLStd	
PiB	100×SPiBUVrIND−1.0141.074	100×SPiBUVrIND−1.0340.993	0.993
FBP	100×SFBPUVrIND−1.0410.561	100×SFBPUVrIND−1.1090.496	0.976
FMM	100×SFMMUVrIND−0.9990.827	100×SFMMUVrIND−1.0590.755	0.991
FBB	100×SFBBUVrIND−1.0060.653	100×SFBBUVrIND−1.0550.599	0.989
NAV	100×SNAVUVrIND−1.0251.123	100×SNAVUVrIND−1.0570.966	0.992

*CTracerLNS vs. CTracerLStd

### 3.2 Level 2 analysis of the non-standard pipeline using PiB

We obtained a slight bias between the PiB SUVr calculated using the non-standard and SPM8 standard pipelines, with a slope (mPiBNS) of 0.924 and an intercept (bPiBNS) of 0.097. The R^2^ was 0.993, indicating that both pipelines were well-correlated. By substituting mPiBNS and bPiBNS into Equation (3), and the resultant SPiB−CalcUVrIND and mean SUVr values into Equation (4), the PiB SUVr using the non-standard pipeline was converted into the Centiloid scale using the following equation (Table 3):

CPiBLNS=100×SPiBUVrIND−1.0340.993

**Fig. 2:**
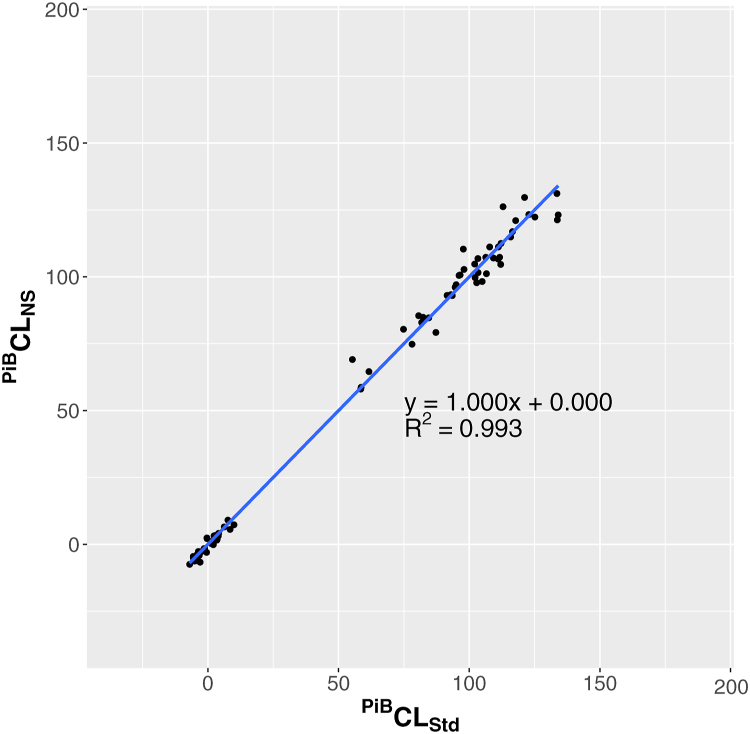
Correlation of the Centiloid scale values from the GAAIN PiB dataset between the SPM8 standard and non-standard pipelines.

The mean Centiloid scale values for the YC-0 and AD-100 subjects were −0.52 and 100.39, respectively. The SD of the Centiloid values for the YC-0 subjects determined using the non-standard pipeline was 4.39, whereas that determined using the SPM8 standard pipeline was 4.41, yielding a relative variance of 1.00 (Table 4). Fig. 2 depicts the correlation of the Centiloid scale values between the non-standard and SPM8 standard pipelines from the GAAIN PiB dataset.

**Table 4:** Relative variances of the Centiloid scale values against $CPiBL_{Std}$ in young controls from each GAAIN dataset.

Dataset	N	CPiBLStd	CPiBLNS	CTracerLStd	CTracerLNS
GAAIN PiB	34				
Mean ± SD		0.00 ± 4.41	−0.52 ± 4.39		
RV		NA	1.00		
GAAIN PiB/FBP	13				
Mean ± SD		−0.67 ± 2.60	2.30 ± 3.59	2.60 ± 9.84	−0.98 ± 12.00
RV		NA	1.38	3.78	4.61
GAAIN PiB/FMM	24				
Mean ± SD		−0.58 ± 6.04	3.05 ± 6.40	−1.10 ± 7.16	−1.72 ± 8.22
RV		NA	1.06	1.19	1.36
GAAIN PiB/FBB	10				
Mean ± SD		−1.12 ± 3.42	−0.50 ± 3.75	0.16 ± 7.01	−3.14 ± 5.91
RV		NA	1.10	2.05	1.73
GAAIN PiB/NAV	10				
Mean ± SD		−2.56 ± 4.26	−0.36 ± 5.23	−2.40 ± 4.30	−2.75 ± 6.38
RV		NA	1.23	1.01	1.50

Abbreviations: N, number; NA, not applicable; RV, relative variance; SD, standard deviation.

### 3.3 Level 2 analysis of the SPM8 standard and non-standard pipelines using ^18^F-labeled tracers

**Fig. 3:**
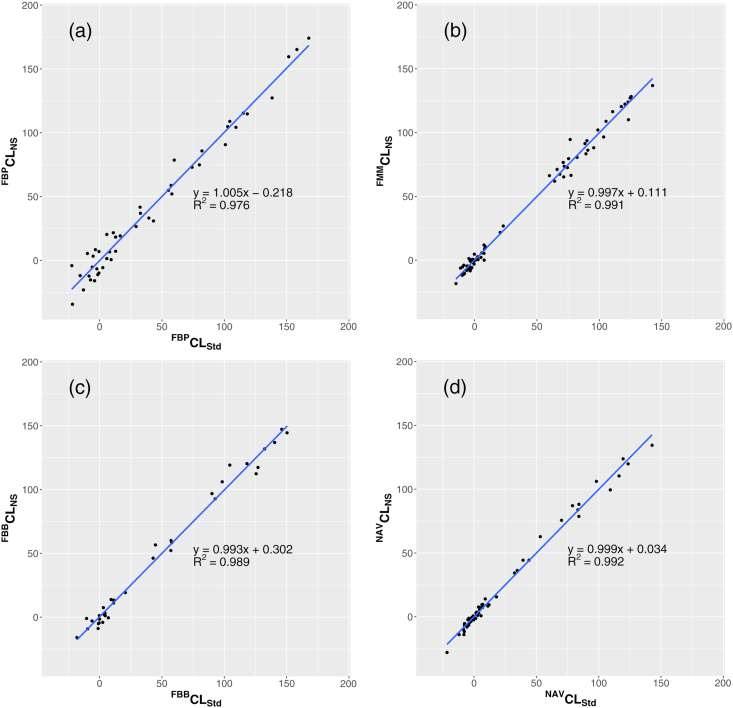
Correlation of the Centiloid scale values from each GAAIN ^18^F-labeled tracer dataset between the SPM8 standard and non-standard pipelines. The x-axis shows the Centiloid values from the SPM8 standard pipeline, and the y-axis shows the Centiloid values from the non-standard pipeline for FBP (a), FMM (b), FBB (c), and NAV (d).

The SUVr values from each ^18^F-labeled tracer by both pipelines were converted into PiB-equivalent SUVr values using the equations depicted in Table 2. The R^2^ values for each correlation were well above the threshold of 0.7 suggested by the Centiloid project paper; in the order of the highest to the lowest, they ranked as follows: NAV, FMM, FBB, and FBP for both pipelines. This was the same as the original paper. The PiB-equivalent SUVr was then converted to the Centiloid scale using the equations shown in Table 3. The R^2^ values for each correlation between the non-standard and SPM8 standard pipelines were very high (>0.97), which was the same as in the original paper. The correlations of the Centiloid scale values between the non-standard and SPM8 standard pipelines using ^18^F-labeled tracers are displayed in Fig. 3. The relative variances of the Centiloid scale values against CPiBLStd using ^18^F-labeled tracers and the non-standard pipeline are described in Table 4 for the young controls from each GAAIN dataset. In the SPM8 standard pipeline, the relative variances of FMM and NAV were 1.19 and 1.01, respectively, which were similar variances to PiB.

In contrast, the relative variances of FBP and FBB were more significant than those of FMM and NAV at 3.78 and 2.05, respectively. These results were comparable to those from previously reported studies (Battle et al., 2018; Navitsky et al., 2018; Rowe et al., 2016, 2017). On the Centiloid scale of PiB using the non-standardized pipeline, the relative variances ranged from 1.00 to 1.38 for each GAAIN dataset. The relative variance in the Centiloid scale of the ^18^F-labeled tracers determined using the non-standard pipeline was the highest for FBP at 4.61, while the other tracers showed values ranging from 1.36 to 1.73 (Table 4).

### 3.4 Validation using datasets from J-ADNI, ADNI, and AIBL

**Fig. 4:**
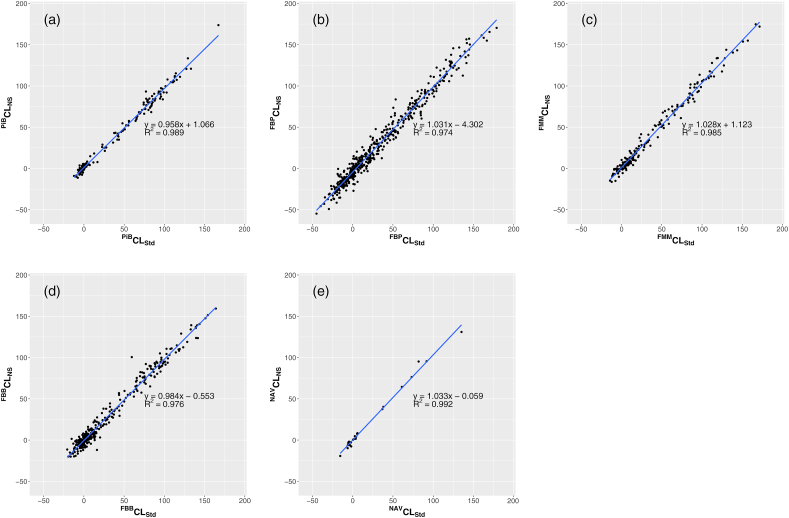
Correlation of the Centiloid scale values from the J-ADNI PiB (a), ADNI FBP (b), AIBL FMM (c), ADNI FBB (d), and AIBL NAV (e) datasets between the SPM8 standard and non-standard pipelines. The x-axis shows the Centiloid values from the SPM8 standard pipeline, and the y-axis shows the Centiloid values from the non-standard pipeline.

The correlations of the Centiloid scale values between the non-standard and SPM8 standard pipelines for the validation datasets, including PiB from J-ADNI, FBP and FBB from ADNI, and FMM and NAV from AIBL, are displayed in Fig. 4. The Centiloid scale values of PiB calculated by the non-standard pipeline showed high agreement with those by the SPM8 standard pipeline (R^2^ = 0.989), and the slope of the regression was underestimated by 4.2%. For FBP, the level of agreement was relatively low (R^2^ = 0.974), but the bias was slight, with a regression slope of 3.1% overestimation. For FMM, a high degree of agreement was found (R^2^ = 0.985), with the regression slope showing an overestimation of 2.8%. For FBB, the agreement was high (R^2^ = 0.976), and the regression slope showed a slight bias, underestimating by 1.6%. Although the number of cases was small for NAV, a very high degree of agreement was found (R^2^ = 0.992), and the regression slope showed an overestimation of 3.3%. A good absolute agreement was observed across all the tracers using the two-way mixed models and single rater unit, with 0.994, 0.986, 0.992, 0.988, and 0.996 for PiB, FBP, FMM, FBB, and NAV, respectively.

## 4 Discussion

In the present study, the Centiloid scale values of amyloid PET computed using the non-standard pipeline without MRI proposed in the original paper (Bourgeat et al., 2018) was shown to have a strong linear relationship with those computed using the SPM8 standard pipeline. This held true even when validation datasets of PiB, FBP, and FBB, which are different from those used in the original paper, were used, suggesting that it is replicable.

The validation datasets of PiB, FBP, FMM, FBB, and NAV all yielded high R^2^ values between the Centiloid scale values calculated by the non-standard and SPM8 standard pipelines. The bias in the FBB and NAV datasets, which was reported to be prominent in the original paper (an underestimation of 11.4% for FBB and an overestimation of 8.1% for NAV), was slight in the present study (an underestimation of 1.6% for FBB and an overestimation of 3.3% for NAV). Since the ADNI FBB dataset (n = 323) used in this study was more prominent than that used in the original paper (n = 119), the estimation of the regression parameters seems more accurate. On the other hand, the AIBL NAV dataset (n = 25) used in this study was even smaller than that used in the original paper (n = 79), limiting the precision of the regression parameter estimates. More data are needed to improve the estimation accuracy. In addition, to evaluate the replicability of the Centiloid scale using the non-standard pipeline, different FMM and NAV validation datasets from those used in the original paper are also needed.

Since the validation datasets used were from J-ADNI for PiB and ADNI for FBP and FBB, there was high heterogeneity in PET scanner manufacturers and models. However, the acquisition protocol and scan parameters, which were uniformly defined for each tracer and scanner model, respectively, and the preprocessing to alleviate differences in PET images from different scanners (Ikari et al., 2012; Joshi et al., 2009) may have contributed to the slight bias. To obtain high agreement and less bias in the Centiloid scale values between the non-standard and SPM8 standard pipelines using different scanner manufacturers or models, standardized image acquisition methods and preprocessing are considered necessary.

**Fig. 5:**
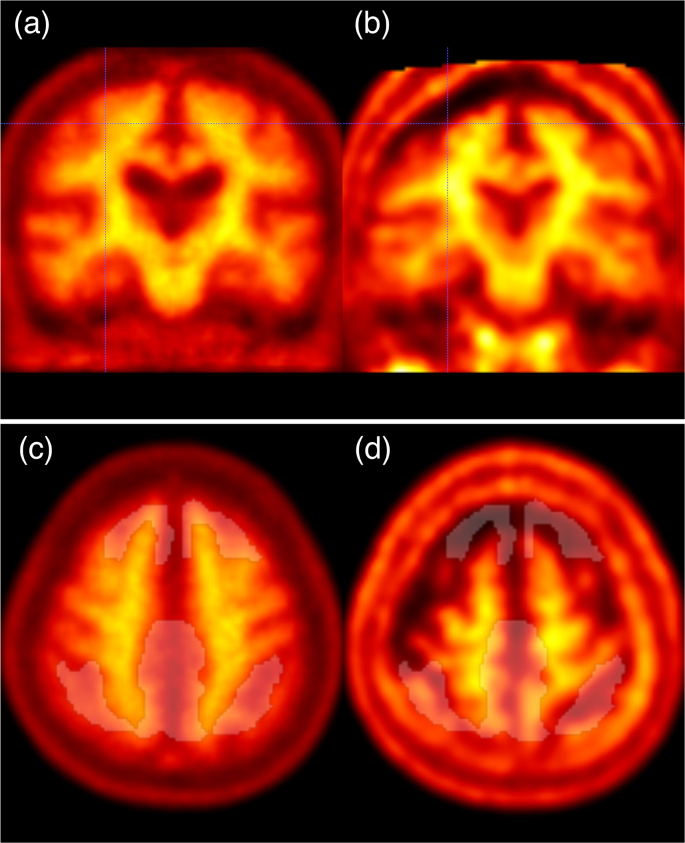
An example of failure in anatomical normalization using the non-standard pipeline. (a) Coronal section of the template image. (b) Anatomically normalized target PET image in the template space. Note that the center of the crosshairs is positioned on the same coordinates as (a). (c) Axial section of the template image with the overlaid translucent white cortical VOI. (d) Axial section of the anatomically normalized target PET image in the template space with the overlaid translucent white cortical VOI. A registration error between the template (a) and nonlinearly registered image (b) is found in the high convexity (the center of the crosshairs). This registration error could be due to the high uptake of a tracer in the diploe and the subcutaneous tissue of the scalp in the target image, leading to failure of cortical VOI extraction (d).

Since both the non-standard and SPM8 standard pipelines measure SUVr values using the common VOIs in the template space, the Centiloid scale value depends on the accuracy of the anatomical normalization. Unlike the SPM8 standard pipeline, which uses structural MRI for anatomical normalization, the non-standard pipeline may be more prone to registration errors because it uses structural information from amyloid PET for anatomical normalization, which has low spatial resolution and provides less anatomical information than MRI. For example, in the scatter plots of the non-standard and SPM8 standard pipelines for the ADNI FBB dataset shown in Fig. 4 (d), there was a positive outlier (CFBBLStd=59.6 vs. CFBBLNS=100.5). Visual assessment reveals that the anatomically normalized image using the non-standard pipeline has a registration error in the high convexity (Fig. 5). This registration error was presumably caused by increased accumulation of FBB in the diploe of the skull and subcortical tissue of the scalp.

**Fig. 6:**
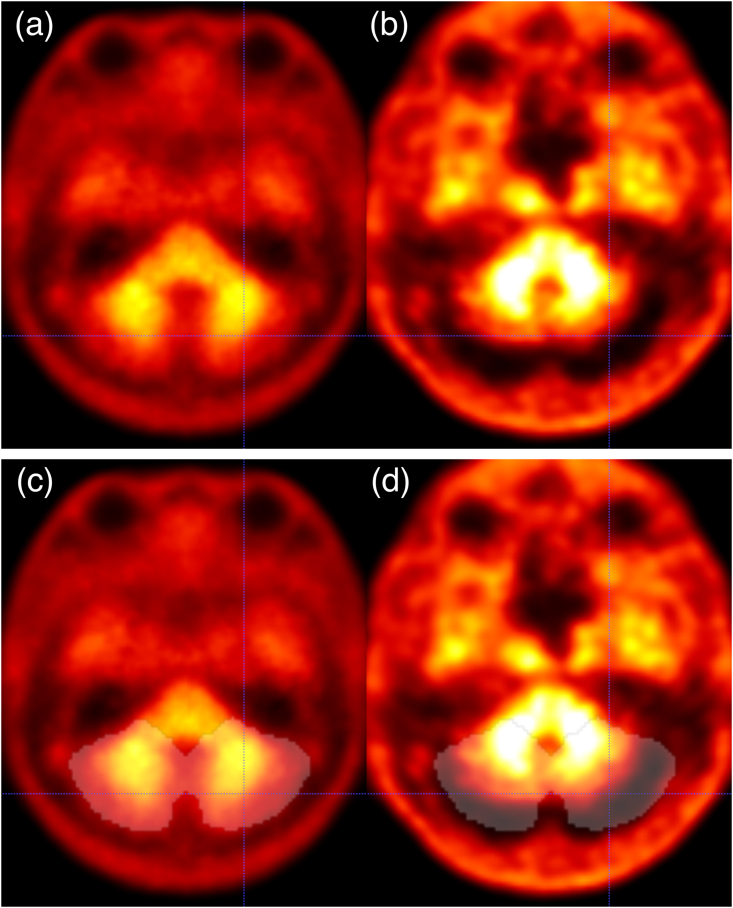
Another example of failure in anatomical normalization using the non-standard pipeline. (a) Axial section of the template. (b) Anatomically normalized target PET image in the template space. Note that the center of the crosshairs is positioned on the same coordinates as (a). (c) Axial section of the template image with the overlaid translucent white whole cerebellar VOI. (d) Axial section of the anatomically normalized target PET image in the template space with the overlaid translucent white whole cerebellar VOI. A registration error between the template (a) and nonlinearly registered image (b) is found in the posterior edge of the bilateral cerebellar hemispheres (the center of the crosshairs). This was due to the expanded cerebrospinal fluid space, leading to failure of whole cerebellar VOI extraction (d).

Another example is the negative outlier (CFBBLStd=16.3 vs. CFBBLNS=−12.0) in the scatter plot of FBB shown in Fig. 4 (d). Visual assessment reveals a dilated cerebrospinal fluid space on the surface of the bilateral cerebellar hemispheres and registration errors in the same areas (Fig. 6). Based on the above, if high accuracy is required even in the presence of an anatomical anomaly, it would be better to prepare structural MRI and use the SPM8 standard pipeline, or if structural MRI cannot be used, to use the pipeline with anatomical normalization using low-dose CT for attenuation correction from PET/CT (Matsuda et al., 2021).

The variance of the Centiloid scale of PiB in young controls with the non-standard pipeline was similar to or slightly larger than that of the SPM8 standard pipeline in the five GAAIN datasets, with low relative variances ranging from 1.00 to 1.38. This finding indicates that the non-standard pipeline can calculate the Centiloid scale value of PiB PET with almost the same or slightly lower accuracy than the standard pipeline. In contrast, when the Centiloid scale was computed using the SPM8 standard pipeline for ^18^F-labeled tracers, the relative variance compared with PiB ranged from 1.01 to 3.78 depending on the tracer, which is consistent with previously reported results (Battle et al., 2018; Navitsky et al., 2018; Rowe et al., 2016, 2017). When the Centiloid scale value was computed using the non-standard pipeline from ^18^F-labeled tracers other than FBB, the relative variance was slightly higher than that computed using the SPM8 standard pipeline, indicating that in addition to the increase in variance with the ^18^F-labeled tracers, the variance with the non-standard pipeline became even more prominent. This finding suggests that combining an ^18^F-labeled tracer and the non-standard pipeline may reduce sensitivity to slight changes in Aβ burden. Among the four ^18^F-labeled tracers, FMM and NAV, which had low relative variances (1.36 for FMM and 1.50 for NAV), may be suitable tracers for calculating the Centiloid scale value using the non-standard pipeline. These lower relative variances are consistent with FMM and NAV having larger slope values (mTracerNS) and broader dynamic ranges than FBP and FBB. FMM and NAV are more similar to PiB in structure than FBP and FBB, suggesting that they have more PiB-like uptake and kinetics.

This study has three potential limitations. First, we used PiB-negative and PiB-positive templates for anatomical normalization of the four ^18^F-labeled tracers in the non-standard pipeline. Each of the four ^18^F-labeled tracers has different imaging characteristics; therefore, a dedicated template for each PET tracer could increase the accuracy of anatomical normalization and result in better agreement between the Centiloid values calculated by the non-standard pipeline and those calculated by the SPM8 standard pipeline. To create a dedicated template for each PET tracer, additional PET and corresponding MRI data must be collected. Second, in this study, we could not examine how different scanner models and reconstruction methods affect the Centiloid values obtained with the non-standard pipeline because the calibration and validation data were acquired using only one identical scanner model for NAV and completely different models for FMM or that the models used for calibration data were rarely used for validation data for FBP and FBB. Third, the PiB-positive template used in the non-standard pipeline was the average of the PET images of 44 PiB-positive subjects; thus, it showed a typical amyloid-positive pattern with high accumulation in the frontal cortex, lateral temporal cortex, inferior parietal cortex, posterior cingulate gyrus, precuneus, and striatum. Therefore, anatomical normalization of PET images that present an amyloid-positive pattern with high accumulation in a few regions may be less accurate than anatomical normalization of images that present the typical amyloid-positive pattern.

## 5 Conclusions

This study showed that the non-standard pipeline without MRI could compute the Centiloid scale value in strong agreement with that computed using the SPM8 standard pipeline with MRI in amyloid PET. It is replicable when using validation datasets different from the original paper. Although more datasets are needed to verify the replicability of FMM and NAV, the low variance of the Centiloid scale values for young controls suggest that FMM and NAV might be suitable in addition to PiB for computing the Centiloid scale values using the non-standard pipeline. Although PET-only anatomical normalization can be vulnerable to anatomical anomalies, use of the non-standard pipeline without MRI might be helpful. This technique may assist in visual assessment of amyloid PET positivity/negativity when structural MRI is not available.

## Disclosures

This research did not receive any specific grant from funding agencies in the public, commercial, or not-for-profit sectors.

## Declaration of competing interest

The authors declare that they have no known competing financial interests or personal relationships that could have appeared to influence the work reported in this paper.
